# Physical diagnoses in nursing home residents - is dementia or severity of dementia of importance?

**DOI:** 10.1186/s12877-018-0943-8

**Published:** 2018-10-22

**Authors:** Live Bredholt Jørgensen, Berit Marie Thorleifsson, Geir Selbæk, Jūratė Šaltytė Benth, Anne-Sofie Helvik

**Affiliations:** 1 Department of Public Health and Nursing, Norwegian University of Science and Technology (NTNU), Trondheim, Norway; 2 Norwegian National Advisory Unit on Ageing and Health, Vestfold Hospital Trust, Tønsberg, Norway; 3 Centre for Old Age Psychiatric Research, Innlandet Hospital Trust, Ottestad, Norway; 4 Institute of Health and Society, Faculty of Medicine, University of Oslo, Oslo, Norway; 5 Institute of Clinical Medicine, University of Oslo, Oslo, Norway; 6 Health Services Research Unit, Akershus University Hospital, Lørenskog, Norway; 7 Department of Public Health and Nursing, Faculty of Medicine and Health Sciences, Norwegian University of Science and Technology (NTNU), Trondheim, Norway; 8 St Olavs University Hospital, Trondheim, Norway

**Keywords:** Dementia, Cognitive impairment, Prevalence, Comorbidity, Multimorbidity, Physical diagnoses, Gender, Nursing home, Institutionalization

## Abstract

**Background:**

Dementia and physical morbidity are primary reasons for nursing home admission globally. However, data on physical morbidity in nursing home residents with and without dementia are scarce. The first aim of the present study was to explore whether presence and severity of dementia were related to the number of physical diagnoses in nursing home residents. The second aim was to explore if the severity of dementia was associated with having registered the most frequent complexes of physical diagnoses when controlling for physical health and demographic factors.

**Methods:**

A total of 2983 Norwegian nursing home residents from two cross-sectional samples from 2004/2005 and 2010/2011 were included in the analysis. By the use of assessment scales, the severity of dementia (Clinical Dementia Rating), physical health (General Medical Health Rating), activities of daily living (Physical Self-Maintenance Scale) and neuropsychiatric symptoms (Neuropsychiatric Inventory Nursing Home) were determined. Physical diagnoses and medications were assembled from the medical records. The physical diagnoses were categorized into complexes, using the ICD-10 chapters. Linear mixed models and generalized linear mixed models were estimated.

**Results:**

Residents with dementia were registered with fewer physical diagnoses than residents without dementia. The frequency of physical diagnoses decreased with increasing severity of dementia. Cardiovascular, musculoskeletal and endocrine, nutritional and metabolic diagnoses were the most common complexes of physical diagnoses in individuals with and without dementia. The odds of having cardiovascular and musculoskeletal diagnoses increased for males and decreased for females with increasing severity of dementia, in contrast to endocrine diagnoses where the odds increased for both genders.

**Conclusion:**

Increasing severity of dementia in nursing home residents may complicate the diagnostics of physical disease. This might reflect a need for more attention to the registration of physical diagnoses in nursing home residents with dementia.

## Background

Dementia is a common disease in aged populations [1] caused by different brain disorders. It results in a decline in memory, especially evident in the learning of new information. Additionally, dementia involves behavioural changes, functional impairment, and a decrease in other cognitive abilities such as thinking, judgement and processing of information [2]. There is a clear link between severity of dementia, impairment in activities of daily living (ADL) [3], the risk of institutionalization [4] and mortality [5].

Worldwide 46.8 million people live with dementia, and the number will almost double every 20 years [6] due to an aging population [7]. In Norway, with a population of about 5 million [8], the calculated number of older adults with dementia was approximately 80,000 in 2015 [9, 10]. Dementia is not the only disease affecting aging individuals to a great extent, as older adults generally have a higher risk of experiencing multiple chronic conditions, both psychiatric and physical [11].

Management of the rising prevalence of chronic conditions is a main challenge facing governments and health-care systems globally [12]. As multimorbidity is becoming the normal situation rather than an exception in the aging population [13–16], it is crucial to focus on physical diagnoses, as well as decreased functional status [17]. Common physical diagnoses in the aging population are hypertension, lipid metabolism disorders, diabetes, coronary heart disease, heart failure and cancer [18–21]. Several of these diseases represent vascular risk factors, which may contribute to dementia onset and lead to faster progression of dementia [22–24]. Parkinson’s disease, congestive heart failure, cerebrovascular disease, cardiac arrhythmia, osteoporosis and retinal disorders [25] are physical comorbidities which seem to be significantly associated with having dementia.

A dementia diagnosis and increasing cognitive impairment are major reasons for nursing home admissions [4, 21]. Residents without dementia are mainly admitted to nursing homes because of severe physical morbidity which makes it difficult for them to continue living at home [26]. Other important factors associated with nursing home admissions are high age, psychosis and increased number of prescriptions [4, 27].

Several international studies have explored the use of psychotropic drugs [28, 29], the prevalence of dementia [30, 31], depression [32, 33] and neuropsychiatric symptoms in nursing homes [33–36]. However, Scandinavian studies exploring physical morbidities in nursing home residents with and without dementia, are to our knowledge missing.

Previous studies have reported a considerable variation in the number of additional diagnoses registered in older adults living with dementia [25, 37, 38]. Studies from primary care found that individuals with dementia had a higher number of comorbidities than those without dementia [25, 39]. On the contrary, nursing home residents with dementia had fewer comorbidities than residents without cognitive impairment or dementia [40, 41]. This might describe the health situation of nursing home residents, but it may also reflect a lack of diagnostics in nursing home residents with dementia that do not complain, have difficulties in describing their symptoms or do not receive frequent clinical examination [20, 24].

Literature regarding physical morbidity in nursing home residents with and without dementia frequently focuses on the most common ICD-10 diagnoses [24, 40–42], but few studies arrange diagnoses by the main ICD-10 chapters. According to published nursing home studies, the most commonly registered physical diagnoses are linked to cardiovascular, musculoskeletal and endocrine diseases [24, 40–42].

Information about physical diagnoses in nursing home residents with and without dementia, and whether such comorbidity is related to the severity of dementia, is essential for healthcare planners and care professionals [24]. Thus, the first aim of the present study was to explore whether presence and severity of dementia were related to the number of physical diagnoses in nursing home residents. The second aim was to explore if the severity of dementia was associated with having registered the most frequent complexes of physical diagnoses when controlling for physical health and demographic factors.

## Methods

### Design

The present study includes data collected from two Norwegian cross-sectional samples of nursing home residents. The first collection took place from November 2004 to January 2005 [43] and the second collection took place from June 2010 to November 2011 [30].

### Participants

**Fig. 1:**
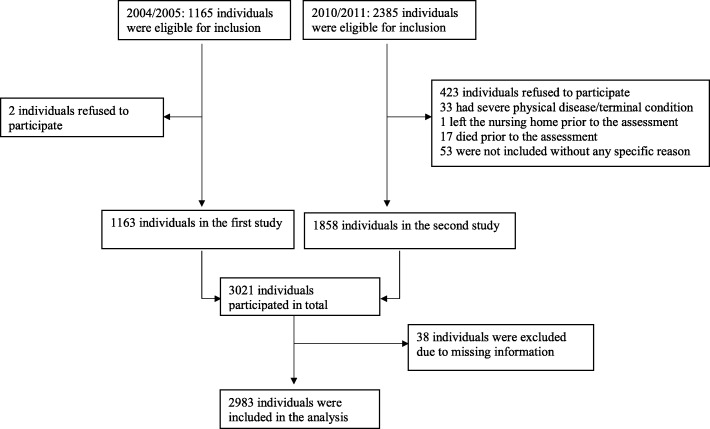
Flow chart of the study population

Both samples included nursing home residents with a stay of minimum 2 weeks [30, 43]. In 2004/2005, residents in 26 nursing homes in 18 municipalities participated, and the selection of municipalities reflected small, medium and large municipalities. A total of 1165 residents were eligible for inclusion, and two refused participation. In 2010/2011, residents from 40 other nursing homes in 31 municipalities were approached in addition to 24 of the 26 nursing homes from the previous sample. A total of 2385 residents were eligible for inclusion, but 423 declined to participate either in person or through their next of kin, 33 had a severe physical diagnosis or terminal condition, one left the nursing home prior to the assessment, 17 died prior to the assessment and 53 were not included without any specific reason. As a result, 1858 participants were included in the second study. In total, 3021 nursing home residents participated in the present study. Thirty-eight residents were excluded due to missing important information (Clinical Dementia Rating), leaving a total of 2983 residents in the analysis (Fig. 1).

### Measurements

All medical diagnoses assembled from the medical records were classified by the International Statistical Classification of Diseases and Related Health Problems 10th Revision (ICD-10). The diagnoses were collected at assessment, based on what was registered in the charts. The charts were regularly updated, so several diagnoses could have been added after admission. Mental behavioural disorders (F00-F99) and Alzheimer’s disease (G30) were omitted to extract only physical diagnoses. The registered physical diagnoses were categorized into complexes of diagnoses, using the ICD-10 chapters. As other authors commonly choose to present single ICD-10 codes, subgroups of the most common ICD-10 codes were included under each complex of physical diagnoses [19, 25, 38, 39]. A minimum of one subgroup was included under each complex.

Dementia and the severity of dementia were determined by using the Clinical Dementia Rating (CDR) scale. The CDR score was determined by healthcare personnel who was the most familiar with the resident, using all available information about the resident. No information was collected directly from their next of kin. CDR assesses six domains of cognitive and functional performing [44]. The categorical score (0, 0.5, 1, 2, 3) is calculated using an algorithm that gives priority to memory [45]. CDR ≥ 1 defines dementia [46, 47]. The categorical scores indicate normal cognitive function (CDR = 0), mild cognitive impairment (CDR = 0.5), mild dementia (CDR = 1), moderate dementia (CDR = 2) and severe dementia (CDR = 3). The sum-score of the six domains (CDR sum of boxes) ranges from 0 to 18, where a higher score indicates more severe dementia. There is a high correlation (≥0.9) between the categorical CDR score and the CDR sum of boxes (CDR-SOB) [48, 49]. The Spearman correlation in the present study was 0.93 [30]. Many of the residents were too frail or mentally impaired to take part in standardized dementia work-up such as CT or MRI. Therefore CDR ≥ 1 was used as an indication of dementia in both samples.

Physical health was assessed using the General Medical Health Rating (GMHR) scale [50]. GMHR is a 1-item global rating scale with four categories: good, fairly good, poor and very poor. All available information about physical health and drug use formed the basis for the rating. GMHR has previously been used in large studies including older adults with and without dementia [51] and has been translated and used in Norway [52].

The Personal Activities of Daily Living (P-ADL) score was assessed with the Physical Self-Maintenance Scale (PSMS), which includes six items and results in a total score ranging from 6 to 30 [53]. A high score indicates a low level of ADL functioning.

Neuropsychiatric symptoms (NPS) were assessed using a translated and validated Norwegian version [54] of the Neuropsychiatric Inventory Nursing Home version (NPI-NH) [55]. The 10-item inventory covers the following symptoms: delusion, hallucination, euphoria, agitation/aggression, disinhibition, irritability/lability, depression/dysphoria, anxiety, apathy/indifference and aberrant motor behaviour (no/yes). Each symptom is graded by severity (score 0–3) and multiplied by frequency (score 0–4), which provides an item-score from 0 to 12. Based on a previous principal component analysis, subsyndrome scores on psychosis (delusions, hallucination), agitation (agitation/aggression, disinhibition, irritability) and affective symptoms (depression, anxiety) were generated [35, 36, 56]. Apathy/indifference was analysed as a single symptom.

Medications were grouped according to the Anatomical Therapeutic Chemical (ATC) classification system. The ATC-system is a classification of the active ingredients of the drugs and is based on the organ or system they act on, and also their pharmacological, therapeutic and chemical characteristics [57]. The information was collected from the medical record of each resident [43].

Demographic information was determined by use of a standardized questionnaire. The type of unit was recorded from the following: regular unit (RU), special care unit for people with dementia (SCU), rehabilitation unit (REU) and other units (OU), mainly psychogeriatric wards.

### Procedure

In both samples, registered nurses with broad clinical experience performed the data collection. All 20 assessors took part in a two-day training course on how to apply the standardized questionnaires prior to the data collection. Data were collected from medical records and a standardized interview with the residents’ primary caregivers. Prior to the first study, a pilot study including 41 nursing home residents was conducted to test the inter-rater reliability of the CDR. It was performed between one geriatric psychiatrist (GP) and two assessors, a registered nurse (RN) and a nurse specialized in psychiatry (NP). The kappa values for the global CDR score were 1 (GP vs. NP) and 0.86 (GP vs. RN and NP vs. RN) [43].

Information about the study was given to the residents and to their family members. An explicit consent was not required for enrolment in 2004/2005, but the residents were informed that they could refuse to participate at any stage of the study. In 2010/2011 informed consent was obtained from the resident or their next of kin due to a change in the legislation. The Regional Ethics Committee in the south-east of Norway and the Directorate for Health and Social Affairs recommended and approved the procedures in 2004 and 2010.

### Statistical analysis

As data were collected in nursing homes, there might be a hierarchical structure in the data. In addition, some of the participants in the first sample (7.7%) were also included in the second sample. A cluster effect might therefore be present at both the nursing home and participant level, and statistical methods that correctly adjust for such an effect have been used.

Means and standard deviations (SD), or frequencies and percentages, were used to present demographic and clinical characteristics. Linear mixed model for continuous variables and generalized linear mixed model for categorical variables were estimated to compare residents with and without dementia. The models included fixed effects for dementia status, and random effects for either participants or nursing homes or both with participants nested within the nursing home, as appropriate.

To explore whether the severity of dementia was related to the number of physical diagnoses and other factors, a linear mixed model with fixed effects for characteristics and random effects for participants nested within nursing homes was estimated. To assess how certain factors affected the odds of having specific complexes of physical diagnoses, a generalized linear mixed model with the same fixed effects was estimated. The model contained random effects for participants only, as cluster effect on the nursing home level was negligible or not present. Interactions between severity of dementia and gender and age were explored. All multiple models were reduced by applying Akaike Information Criterion (AIC), where the smaller value indicates a better model. In post hoc analysis for factors associated with the number of physical diagnoses and the three most prevalent complexes of physical diagnoses, the GMHR was included to explore whether the level of general medical health influenced an association between level of dementia and the number of physical diagnoses.

Analyses were performed in SPSS v 24 and SAS v 9.4. All statistical tests were two-sided. Results with *p*-values below 0.05 were considered statistically significant.

## Results

### Sample characteristics at baseline

The present study included 2983 nursing home residents assessed at two different time-points. In total, 808 residents lived in special care units and 2164 residents lived in other units. Of all participants, 82.8% had dementia (CDR ≥ 1) (Table 1). Among those without dementia (CDR < 1), 81.3% had mild cognitive impairment (CDR = 0.5). Mean (SD) age was 85.1 (7.9) years and 71.5% were females. Individuals with dementia were older than those without dementia. They were also more likely to have a poorer physical health (GMHR), poorer P–ADL functioning (higher PSMS score), higher scores on the NPI subsyndromes agitation, psychosis and affective, and NPI apathy, a longer stay in the nursing home at study inclusion, and to be registered with a lower mean number of drugs.

**Table 1:** Sample characteristics at baseline

		Total	CDR < 1	CDR ≥ 1	*P*-value^3^
	N (%)	2983 (100)	513 (17.2)	2470 (82.8)	
Sociodemographics					
Age	Mean (SD)	85.1 (7.9)	84.3 (9.2)	85.3 (7.6)	0.019^1^
Females	N (%)	2132 (71.5)	352 (68.6)	1780 (72.1)	0.137^1^
Education < 10 years	N (%)	2227 (79.7)	376 (78.7)	1851 (80.0)	0.173^2^
Married	N (%)	630 (21.1)	86 (16.9)	544 (22.2)	0.018^2^
Health condition					
GMHR					< 0.001^2^
Good	N (%)	474 (16.1)	101 (20.1)	373 (15.3)	
Fairly good	N (%)	1097 (37.3)	220 (43.7)	877 (36.0)	
Poor	N (%)	1033 (35.1)	142 (28.2)	891 (36.6)	
Very poor	N (%)	335 (11.4)	40 (8.0)	295 (12.1)	
PSMS score	Mean (SD)	18.0 (5.4)	14.9 (5.0)	18.6 (5.2)	< 0.001^1^
NPI Agitation subsyndrome	Mean (SD)	6.0 (8.2)	2.3 (5.1)	6.7 (8.5)	< 0.001^1^
NPI Psychosis subsyndrome	Mean (SD)	2.7 (5.1)	0.9 (3.2)	3.1 (5.3)	< 0.001^1^
NPI Affective subsyndrome	Mean (SD)	3.5 (5.2)	2.4 (4.3)	3.7 (5.3)	< 0.001^1^
NPI Apathy	Mean (SD)	2.0 (3.5)	0.8 (2.3)	2.3 (3.7)	< 0.001^1^
Number of drugs	Mean (SD)	6.6 (3.2)	7.7 (3.6)	6.4 (3.1)	< 0.001^1^
Days in NH^4^	Mean (SD)	931.0 (997.9)	882.5 (1162.8)	941.1 (960.0)	< 0.001^1^

*CDR* Clinical Dementia Rating, *GMHR* General Medical Health Rating, *PSMS* Physical Self-Maintenance Scale, *NPI* Neuropsychiatric Inventory, *NH* Nursing Home

^1^Adjusted for intra-patient correlations

^2^Adjusted for NH-level

^3^Calculated by estimating linear mixed model for continuous variables and generalized linear mixed model for categorical variables

^4^*p*-value calculated on LN-transformed days in NH

### Factors associated with increasing number of physical diagnoses

Residents without dementia had a higher mean number of physical diagnoses registered than residents with dementia (2.9 versus 2.4) (Table 2). According to the adjusted linear mixed model, residents in special care units were registered with a lower number of physical diagnoses, compared to residents in regular units. Furthermore, lower CDR-SOB, higher age and higher PSMS score were associated with having a higher number of physical diagnoses (Table 3).

**Table 2:** Number of physical diagnoses

Number of physical diagnoses		Total	CDR < 1	CDR ≥ 1	*P*-value
0	N (%)	341 (11.4)	24 (4.7)	317 (12.8)	< 0.001^1^
1	N (%)	607 (20.3)	82 (16.0)	525 (21.3)	
2	N (%)	693 (23.2)	127 (24.8)	566 (29.9)	
3	N (%)	601 (20.1)	113 (22.0)	488 (19.8)	
4	N (%)	374 (12.5)	76 (14.8)	298 (12.1)	
5	N (%)	176 (5.9)	50 (9.7)	126 (5.1)	
Over or equal to 6	N (%)	191 (6.4)	41 (8.0)	150 (6.1)	
Mean number of diagnoses	Mean (SD)	2.5 (1.7)	2.9 (1.8)	2.4 (1.7)	

*CDR* Clinical Dementia Rating

^1^Adjusted for NH-level

**Table 3:** Factors associated with number of physical diagnoses

Variables	Unadjusted		Adjusted	
	Regression coefficient (95% CI)	*p*-value	Regression coefficient (95% CI)	*p*-value
CDR-SOB	−0.06 (− 0.07; − 0.05)	< 0.001	−0.07 (− 0.08; − 0.05)	< 0.001
Males	0.03 (− 0.12; 0.17)	0.720	0.02 (− 0.12; 0.17)	0.734
Age	0.02 (0.01; 0.03)	< 0.001	0.02 (0.01; 0.03)	< 0.001
Education (≥10 years)	−0.18 (− 0.35; − 0.02)	0.025		
PSMS score	−0.001 (− 0.01; 0.01)	0.836	0.03 (0.02; 0.05)	< 0.001
NPI Agitation subsyndrome	−0.02 (− 0.03; − 0.01)	< 0.001		
NPI Psychosis subsyndrome	−0.03 (− 0.04; − 0.02)	< 0.001		
NPI Affective subsyndrome	− 0.01 (− 0.02; 0.005)	0.236		
NPI Apathy	−0.03 (− 0.05; − 0.01)	0.003		
Duration in NH (LN)	−0.04 (− 0.10; 0.01)	0.112		
Type of NH unit				
Regular – ref.	0	–	0	–
Special care	−0.73 (− 0.87; − 0.58)	< 0.001	−0.44 (− 0.59; − 0.28)	< 0.001
Rehabilitation	0.40 (0.01; 0.79)	0.042	0.35 (− 0.04; 0.73)	0.079
Other	−0.12 (− 0.39; 0.14)	0.361	0.07 (− 0.20; 0.34)	0.604

Unadjusted and adjusted analyses using linear mixed model

*CDR-SOB* Clinical Dementia Rating - Sum of Boxes, *PSMS* Physical Self-Maintenance Scale, *NPI* Neuropsychiatric Inventory, *NH* Nursing Home, *LN* Natural Logarithm

### Complexes and subgroups of physical diagnoses by dementia (CDR < 1/CDR ≥ 1) and increasing severity of dementia (CDR)

The most frequent complexes of physical diagnoses in individuals with and without dementia were cardiovascular (60.3%), musculoskeletal (23.7%) and endocrine, nutritional and metabolic diagnoses (22.2%) (Table 4). Cardiovascular diagnoses, musculoskeletal diagnoses, respiratory diagnoses and cancer were more frequent in individuals without dementia compared to individuals with dementia. Of the subgroups, cerebrovascular disease, heart failure, inflammatory joint disease and asthma/chronic obstructive pulmonary disease (COPD) were more common in residents without dementia. The prevalence of respiratory diagnoses, and cardiovascular diagnoses such as hypertension, cerebrovascular disease, ischemic heart disease, arrhythmia and heart failure, decreased with increasing CDR (Table 4).

**Table 4:** Frequency of complexes and subgroups of physical diagnoses by dementia and increasing severity of dementia

Physical diagnoses	N (%)	Total	CDR < 1	CDR ≥ 1	*P*-value	CDR 1	CDR 2	CDR 3	*P*-value
		2983 (100)	513 (17.2)	2470 (82.8)		543 (22.0)	835 (33.8)	1092 (44.2)	
Cardiovascular diagnoses	N (%)	1798 (60.3)	343 (66.9)	1455 (58.9)	0.001^1^	366 (67.4)	521 (62.4)	568 (52.0)	< 0.001^1^
Hypertension (I10–15)	N (%)	662 (22.2)	121 (23.6)	541 (21.9)	0.315^1^	126 (23.2)	206 (24.7)	209 (19.1)	0.030^1^
Cerebrovascular disease (I60–69)	N (%)	642 (21.5)	130 (25.3)	512 (20.7)	0.026^2^	139 (25.6)	179 (21.4)	194 (17.8)	0.001^2^
Ischemic heart disease (I20–25)	N (%)	466 (15.6)	93 (18.1)	373 (15.1)	0.099^2^	95 (17.5)	137 (16.4)	141 (12.9)	0.011^2^
Arrhythmia (I44–49)	N (%)	404 (13.5)	82 (16.0)	322 (13.0)	0.084^2^	83 (15.3)	111 (13.3)	128 (11.7)	0.049^2^
Heart failure (I50)	N (%)	389 (13.0)	93 (18.1)	296 (12.0)	< 0.001^2^	88 (16.2)	92 (11.0)	116 (10.6)	0.005^2^
Musculoskeletal diagnoses	N (%)	707 (23.7)	153 (29.8)	554 (22.4)	0.001^1^	126 (23.2)	201 (24.1)	227 (20.8)	0.274^1^
Osteoporosis (M80–81)	N (%)	265 (8.9)	57 (11.1)	208 (8.4)	0.077^1^	43 (7.9)	80 (9.6)	85 (7.8)	0.892^1^
Arthrosis (M15–19)	N (%)	237 (7.9)	45 (8.8)	192 (7.8)	0.557^1^	43 (7.9)	62 (7.4)	87 (8.0)	0.815^1^
Inflammatory joint disease (M05–14)	N (%)	137 (4.6)	35 (6.8)	102 (4.1)	0.011^2^	26 (4.8)	38 (4.6)	38 (3.5)	0.181^2^
Endocrine, nutritional and metabolic diagnoses	N (%)	662 (22.2)	112 (21.8)	550 (22.3)	0.838^2^	124 (22.8)	187 (22.4)	239 (21.9)	0.661^2^
Diabetes (E10–14)	N (%)	455 (15.3)	83 (16.2)	372 (15.1)	0.530^2^	86 (15.8)	130 (15.6)	156 (14.3)	0.380^2^
Disorders of the thyroid gland (E00–07)	N (%)	195 (6.5)	30 (5.8)	165 (6.7)	0.494^2^	43 (7.9)	51 (6.1)	71 (6.5)	0.380^2^
Neurological diagnoses	N (%)	464 (15.6)	92 (17.9)	372 (15.1)	0.109^2^	89 (16.4)	108 (12.9)	175 (16.0)	0.810^2^
Parkinson (G20)	N (%)	137 (4.6)	32 (6.2)	105 (4.3)	0.057^1^	25 (4.6)	30 (3.6)	50 (4.6)	0.845^1^
Transient ischemic attack (TIA) (G45.9)	N (%)	119 (4.0)	16 (3.1)	103 (4.2)	0.253^1^	16 (2.9)	36 (4.3)	51 (4.7)	0.107^1^
Respiratory diagnoses	N (%)	271 (9.1)	66 (12.9)	205 (8.3)	0.002^2^	57 (10.5)	77 (9.2)	71 (6.5)	0.006^2^
Asthma/COPD (J40–47)	N (%)	242 (8.1)	60 (11.7)	182 (7.4)	0.002^2^	46 (8.5)	70 (8.4)	66 (6.0)	0.054^2^
Genitourinal diagnoses	N (%)	267 (9.0)	52 (10.1)	215 (8.7)	0.312^2^	42 (7.7)	66 (7.9)	107 (9.8)	0.123^2^
Renal failure (N17–19)	N (%)	95 (3.2)	23 (4.5)	72 (2.9)	0.078^2^	18 (3.3)	24 (2.9)	30 (2.7)	0.552^2^
Malign neoplasms	N (%)	249 (8.3)	62 (12.1)	187 (7.6)	0.001^1^	42 (7.7)	70 (8.4)	75 (6.9)	0.406^1^
Malignant neoplasm of breast (C50)	N (%)	54 (1.8)	9 (1.8)	45 (1.8)	0.920^1^	10 (1.8)	15 (1.8)	20 (1.8)	0.999^1^
Gastrointestinal diagnoses	N (%)	230 (7.7)	36 (7.0)	194 (7.9)	0.379^1^	52 (9.6)	67 (8.0)	75 (6.9)	0.068^1^
Ulcer (oesophagus, stomach and duodenum) (K25–28)	N (%)	65 (2.2)	10 (1.9)	55 (2.2)	0.716^2^	10 (1.8)	21 (2.5)	24 (2.2)	0.781^2^
Other									
Fracture of the femur (S72)	N (%)	261 (8.7)	49 (9.6)	212 (8.6)	0.833^1^	54 (9.9)	65 (7.8)	93 (8.5)	0.651^1^
Cataract (H25–26)	N (%)	138 (4.6)	32 (6.2)	106 (4.3)	0.065^2^	19 (3.5)	35 (4.2)	52 (4.8)	0.238^2^
Glaucoma (H40–42)	N (%)	101 (3.4)	11 (2.1)	90 (3.6)	0.100^2^	25 (4.6)	21 (2.5)	44 (4.0)	0.890^2^

*CDR* Clinical Dementia Rating, *COPD* Chronic Obstructive Pulmonary Disease

^1^Adjusted for NH-level

^2^Adjusted for intra-patient correlation

### Factors associated with the three most prevalent complexes of physical diagnoses

**Fig. 2:**
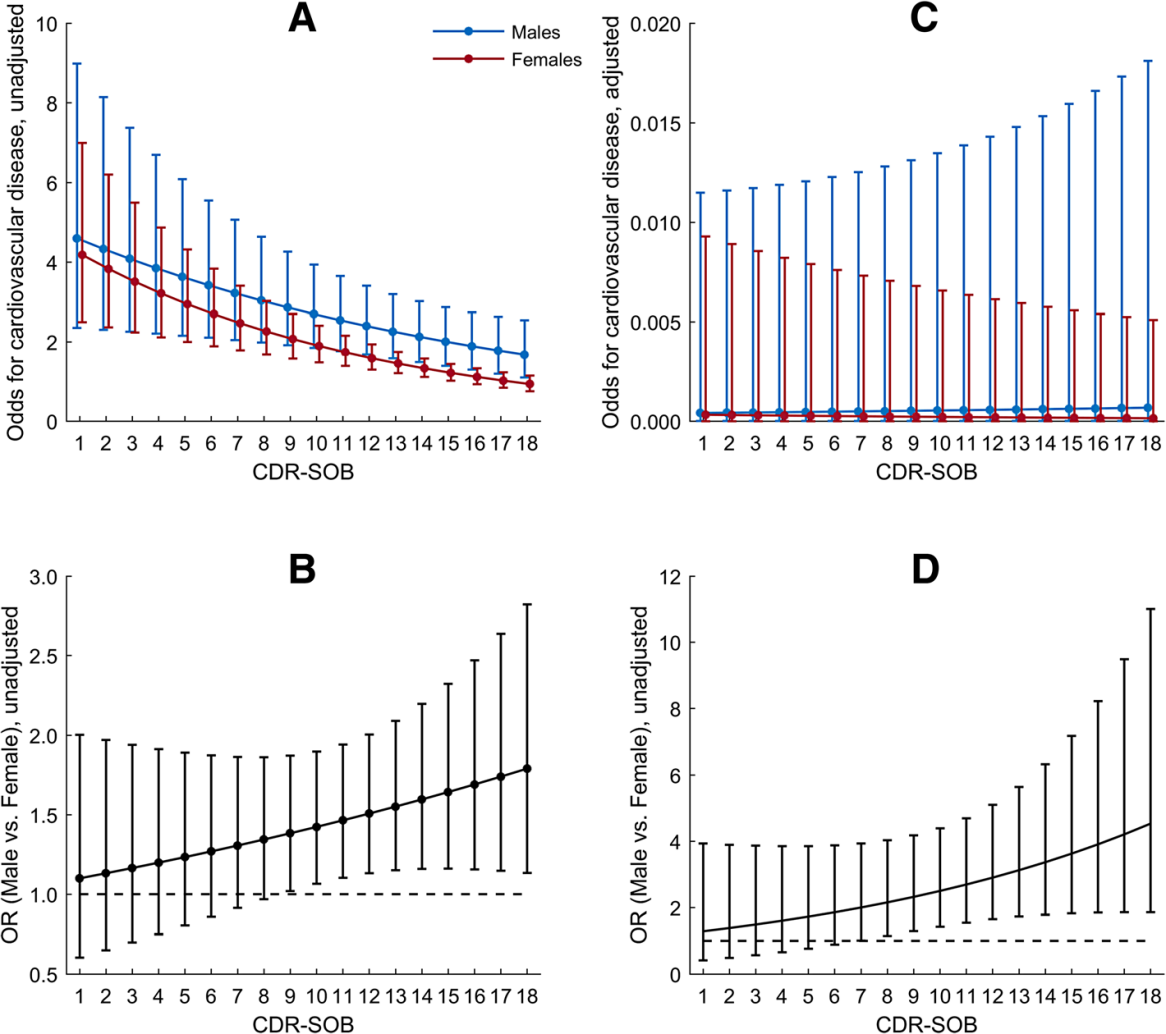
Interpreting interaction term CDR-SOB x Females in Table 5; unadjusted odds for cardiovascular disease (**a**), adjusted odds for cardiovascular disease (**c**), unadjusted OR for Males vs. Females (**b**), and adjusted OR for Males vs. Females (**d**)

Cardiovascular diagnoses were the most frequently registered complex of physical diagnoses in the present study. According to the adjusted generalized linear mixed model analysis, older age, less severe NPI agitation and a higher total number of physical diagnoses were associated with higher odds of having cardiovascular diagnoses (Table 5). Also, an interaction between gender and CDR-SOB was found. In the unadjusted analysis, the odds for cardiovascular disease were decreasing with increasing values of CDR-SOB for both genders, and the reduction was slightly faster for females (Fig. 2a, b). However, in the adjusted analysis, the odds for cardiovascular disease were decreasing for females and increasing for males with increasing values of CDR-SOB (Fig. 2c). For a 1-unit increase in CDR-SOB, the odds for cardiovascular disease were increasing by 8 % more in males than females (OR = 1.08; 95% CI, 0.98–1.19; *p* = 0.142) (Fig. 2d). The odds became significantly different in males versus females for CDR-SOB values above six.

**Table 5:** Factors associated with cardiovascular diagnoses

Variables	Unadjusted		Adjusted	
	OR (95% CI)	*p*-value	OR (95% CI)	*p*-value
CDR-SOB	0.92 (0.89; 0.95)	< 0.001	−0.05 (0.03)^1^	0.100
Males	1.64 (1.17; 2.28)	0.005	0.17 (0.62)^1^	0.783
Age	1.06 (1.03; 1.09)	< 0.001	1.07 (1.04; 1.11)	< 0.001
Education (≥10 years)	0.66 (0.46; 0.93)	0.019	0.66 (0.39; 1.14)	0.144
PSMS score	0.98 (0.96; 1.00)	0.094		
NPI Agitation subsyndrome	0.96 (0.94; 0.98)	< 0.001	0.97 (0.94; 0.99)	0.023
NPI Psychosis subsyndrome	0.95 (0.93; 0.98)	0.001		
NPI Affective subsyndrome	0.97 (0.94; 0.99)	0.014		
NPI Apathy	0.93 (0.90; 0.97)	0.001		
Number of physical diagnoses	8.41 (4.63; 15.28)	< 0.001	8.10 (4.56; 14.37)	< 0.001
Duration in NH (LN)	0.88 (0.79; 0.98)	0.022	0.84 (0.70; 1.02)	0.078
Type of NH unit				
Regular – ref.	1	–		
Special care	0.33 (0.21; 0.52)	< 0.001		
Rehabilitation	1.30 (0.59; 2.86)	0.516		
Other	0.88 (0.52; 1.48)	0.620		
CDR-SOB x Females			0.07 (0.05)^1^	0.142

Unadjusted and adjusted analyses using generalized linear mixed model

*CDR-SOB* Clinical Dementia Rating - Sum of Boxes, *PSMS* Physical Self-Maintenance Scale, *NPI* Neuropsychiatric Inventory, *NH* Nursing Home, *LN* Natural Logarithm

^1^Regression coefficient (standard error) as the OR has no interpretation for interaction and variables included into interaction term

**Fig. 3:**
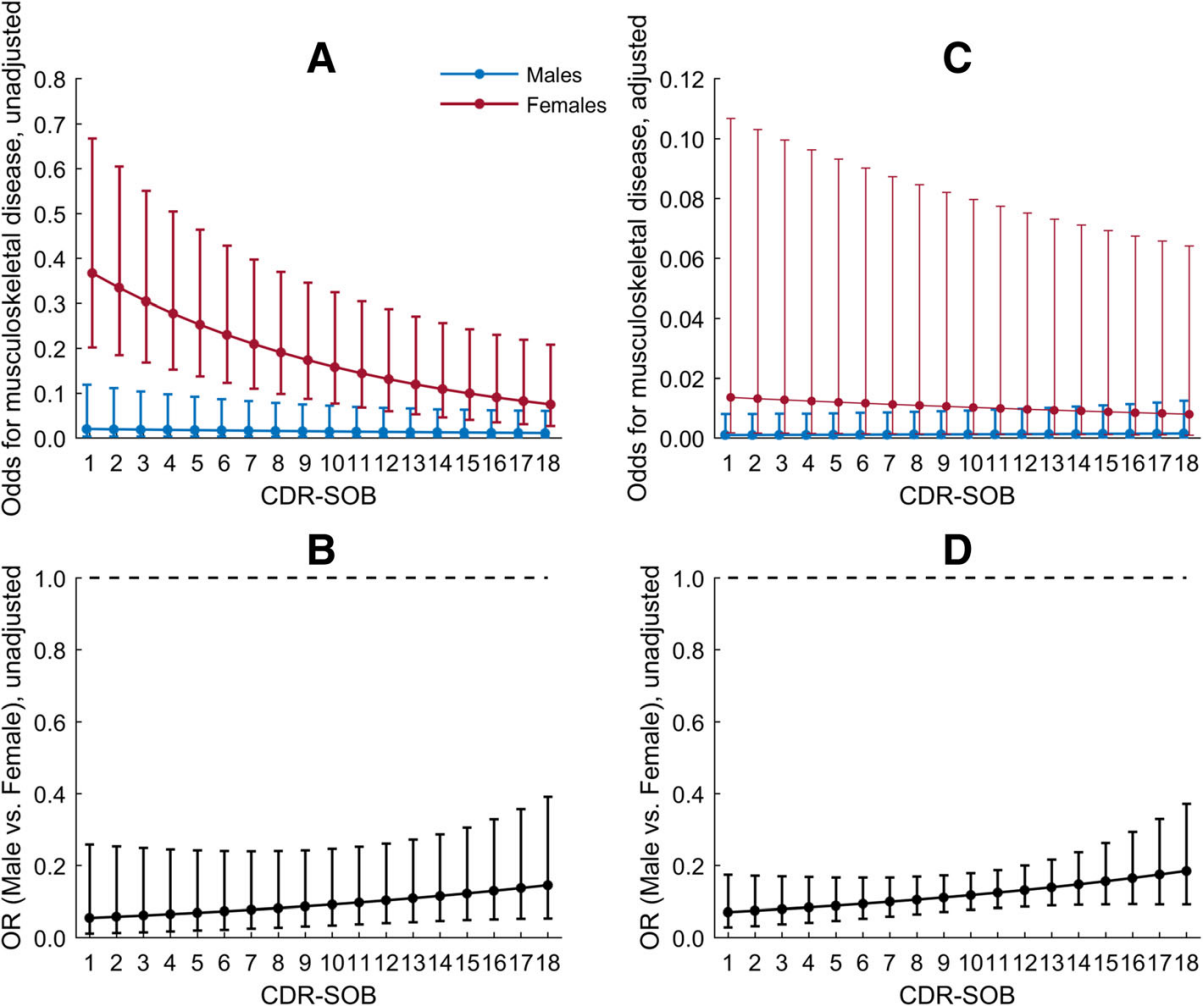
Interpreting interaction term CDR-SOB x Females in Table 7; unadjusted odds for musculoskeletal disease(**a**), adjusted odds for musculoskeletal disease (**c**), unadjusted OR for Males vs. Females (**b**), and adjusted OR for Males vs. Females (**d**)

In the adjusted generalized linear mixed analysis for musculoskeletal diagnoses, factors associated with greater odds were old age, female gender and a higher number of physical diagnoses (Table 6). No interactions were present in the model. The only post hoc analysis being affected by the inclusion of GMHR was post hoc analysis for musculoskeletal diagnoses. Fairly good as compared to good GMHR, shorter duration in a nursing home, longer education and a higher number of physical diagnoses were associated with higher odds of having musculoskeletal diagnoses (Table 7). Furthermore, an interaction between gender and CDR-SOB was detected. In unadjusted analysis, the odds of having musculoskeletal diagnoses were decreasing with increasing values of CDR-SOB for both genders, but the reduction was more pronounced for females (Fig. 3a, b). In the adjusted model, the odds were slightly decreasing for females and increasing for males with increasing values of CDR-SOB (Fig. 3c). For a 1-unit increase in CDR-SOB, males had 6 % higher odds compared to females (OR = 1.06; 95% CI, 0.98–1.15, *p* = 0.174), but the odds were significantly lower than one for all CDR-SOB values (Fig. 3d).

**Table 6:** Factors associated with musculoskeletal diagnoses

Variables	Unadjusted		Adjusted	
	OR (95% CI)	*p*-value	OR (95% CI)	*p*-value
CDR-SOB	0.94 (0.90; 0.97)	0.002	0.97 (0.93; 1.02)	0.220
Males	0.10 (0.04; 0.26)	< 0.001	0.08 (0.02; 0.27)	< 0.001
Age	1.07 (1.03; 1.11)	< 0.001	1.05 (1.01; 1.08)	0.012
Education (≥10 years)	1.04 (0.60; 1.79)	0.888	1.69 (0.99; 2.90)	0.061
PSMS score	0.97 (0.93; 0.99)	0.039		
NPI Agitation subsyndrome	0.97 (0.94; 0.99)	0.006		
NPI Psychosis subsyndrome	0.96 (0.93; 0.99)	0.044		
NPI Affective subsyndrome	1.01 (0.97; 1.05)	0.594		
NPI Apathy	0.93 (0.88; 0.99)	0.018		
Number of physical diagnoses	2.86 (2.15; 3.81)	< 0.001	2.63 (1.66; 4.17)	< 0.001
Duration in NH (LN)	0.88 (0.74; 1.05)	0.165	0.86 (0.71; 1.04)	0.121
Type of NH unit				
Regular – ref.	1	–		
Special care	0.67 (0.44; 1.02)	0.064		
Rehabilitation	1.71 (0.62; 4.73)	0.298		
Other	1.34 (0.67; 2.70)	0.403		

Unadjusted and adjusted analyses using generalized linear mixed model. No significant interactions found in the adjusted model

*CDR-SOB* Clinical Dementia Rating - Sum of Boxes, *PSMS* Physical Self-Maintenance Scale, *NPI* Neuropsychiatric Inventory, *NH* Nursing Home, *LN* Natural Logarithm

**Table 7:** Factors associated with musculoskeletal diagnoses

Variables	Unadjusted		Adjusted	
	OR (95% CI)	*p*-value	OR (95% CI)	*p*-value
CDR-SOB	0.94 (0.90; 0.97)	0.002	−0.03 (0.02)^1^	0.092
Males	0.10 (0.04; 0.26)	< 0.001	−2.71 (0.50)^1^	< 0.001
Age	1.07 (1.03; 1.11)	< 0.001	1.02 (0.99; 1.04)	0.130
Education (≥10 years)	1.04 (0.60; 1.79)	0.888	1.68 (1.12; 2.52)	0.014
GMHR				
Good – ref.	1	–	1	–
Fairly good	3.18 (1.63; 6.18)	0.001	1.72 (1.05; 2.82)	0.037
Poor	1.95 (1.07; 3.55)	0.030	1.11 (0.66; 1.87)	0.703
Very poor	2.00 (0.94; 4.24)	0.070	1.23 (0.63; 2.40)	0.542
PSMS score	0.97 (0.93; 0.99)	0.039		
NPI Agitation subsyndrome	0.97 (0.94; 0.99)	0.006		
NPI Psychosis subsyndrome	0.96 (0.93; 0.99)	0.044		
NPI Affective subsyndrome	1.01 (0.97; 1.05)	0.594		
NPI Apathy	0.93 (0.88; 0.99)	0.018		
Number of physical diagnoses	2.86 (2.15; 3.81)	< 0.001	2.16 (1.96; 2.38)	< 0.001
Duration in NH (LN)	0.88 (0.74; 1.05)	0.165	0.87 (0.76; 0.99)	0.044
Type of NH unit				
Regular – ref.	1	–		
Special care	0.67 (0.44; 1.02)	0.064		
Rehabilitation	1.71 (0.62; 4.73)	0.298		
Other	1.34 (0.67; 2.70)	0.403		
CDR-SOB x Females			0.06 (0.04)^1^	0.174

Unadjusted and adjusted analyses using generalized linear mixed model. GMHR included as explanatory variable

*CDR-SOB* Clinical Dementia Rating - Sum of Boxes, *GMHR* General Medical Health Rating, *PSMS* Physical Self-Maintenance Scale, *NPI* Neuropsychiatric Inventory, *NH* Nursing Home, *LN* Natural Logarithm

^1^Regression coefficient (standard error) as the OR has no interpretation for interaction and variables included into interaction term

Factors associated with higher odds of having endocrine, nutritional or metabolic diagnoses in the adjusted generalized linear mixed analysis were a higher score in CDR-SOB, a greater NPI agitation score, younger age and a higher number of physical diagnoses (Table 8).

**Table 8:** Factors associated with endocrine, nutritional and metabolic diagnoses

Variables	Unadjusted		Adjusted	
	OR (95% CI)	*p*-value	OR (95% CI)	*p*-value
CDR-SOB	0.98 (0.93; 1.02)	0.302	1.07 (1.00; 1.14)	0.049
Males	0.75 (0.42; 1.32)	0.313	0.71 (0.40; 1.26)	0.241
Age	0.99 (0.96; 1.02)	0.588	0.96 (0.92; 0.99)	0.009
Education (≥10 years)	0.54 (0.29; 1.02)	0.056	0.62 (0.33; 1.18)	0.152
PSMS score	0.99 (0.95; 1.04)	0.770	0.94 (0.89; 1.00)	0.053
NPI Agitation subsyndrome	1.02 (0.99; 1.05)	0.334	1.04 (1.00; 1.07)	0.035
NPI Psychosis subsyndrome	0.99 (0.95; 1.04)	0.773		
NPI Affective subsyndrome	1.01 (0.96; 1.06)	0.663		
NPI Apathy	0.99 (0.92; 1.06)	0.663		
Number of physical diagnoses	3.69 (2.82; 4.82)	< 0.001	3.90 (2.95; 5.15)	< 0.001
Duration in NH (LN)	1.07 (0.88; 1.30)	0.509		
Type of NH unit				
Regular – ref.	1	–		
Special care	0.94 (0.53; 1.67)	0.828		
Rehabilitation	2.43 (0.55; 10.77)	0.237		
Other	2.00 (0.73; 5.5)	0.176		

Unadjusted and adjusted analyses using generalized linear mixed model

*CDR-SOB* Clinical Dementia Rating - Sum of Boxes, *PSMS*: Physical Self-Maintenance Scale, *NPI* Neuropsychiatric Inventory, *NH* Nursing Home, *LN* Natural Logarithm

## Discussion

### Main findings

In the present study, the mean number of physical diagnoses registered was lower among nursing home residents with dementia than among those without. The number of physical diagnoses registered decreased with increasing severity of dementia. Cardiovascular, musculoskeletal and endocrine, nutritional and metabolic diagnoses were the three most frequently registered complexes of physical diagnoses.

Cardiovascular diagnoses were more frequently registered in residents without dementia than residents with dementia. Increasing severity of dementia in female residents reduced the odds of having cardiovascular diagnoses, while in male residents the odds increased to some degree. Also, for musculoskeletal diagnoses, the odds slightly decreased for females and increased for males with increasing severity of dementia. For endocrine, nutritional and metabolic diagnoses, the odds increased with severity of dementia for both genders.

### Factors associated with the number of physical diagnoses in nursing home residents

Nursing home residents in the present study had a mean number of 2.5 physical diagnoses registered at inclusion. In studies from long-term care facilities, the total number of diagnoses has been reported to be between 3.0 to 6.4, but these studies did not separate physical diagnoses from mental and behavioural diagnoses [40–42]. A comparison is difficult due to differences in the type of diagnoses registered, sample inequalities and the research methods used. We may speculate that inequalities in health care systems can explain some of the differences. For instance, in the U.S., health care facilities may be financed by insurances [58, 59], which links the number of diagnoses registered closely to economy. Conversely, health care in Norway is mostly publicly financed [59], and nursing home cost is related to registered functional needs.

In the present study, a higher number of physical diagnoses was associated with lower severity of dementia, a higher age, poorer physical health and type of nursing home unit. The number of physical diagnoses registered was higher in residents without dementia. This finding could imply that residents without dementia are admitted to nursing homes because of severe physical morbidity, while individuals with dementia are admitted mainly because of cognitive impairment [4, 26]. We can also speculate that residents with cognitive impairment receive less attention to physical symptoms, which may cause undiagnosed physical disease. However, residents with dementia were more likely to have poorer physical health and poorer P-ADL functioning, which have been found to correlate to a higher number of comorbidities [50]. This could imply that residents with dementia have more comorbid conditions than registered.

Furthermore, increasing severity of dementia was associated with a decrease in the number of physical diagnoses registered. Previous literature from nursing homes have similar findings [40]. In individuals with severe dementia, the accompanying neuropsychiatric symptoms may become clinically dominant and detract attention from other conditions [60]. Moreover, the ability to express physical symptoms and pain is reduced with advanced dementia [61]. Additionally, confusion, agitation and behavioural changes are symptoms that can be interpreted as either symptoms of dementia or physical disease [62]. Finally, the present study also revealed a lower number of physical diagnoses registered in residents in special care units compared to regular care units. The severity of neuropsychiatric symptoms in individuals with dementia is a principal reason for admission to special care units [34]. Thus, careful examination is essential to differentiate between symptoms of delirium, often representing severe underlying physical disease, and neuropsychiatric symptoms associated with dementia [63].

### Factors associated with the three most frequent complexes of physical diagnoses

Cardiovascular, musculoskeletal and endocrine, nutritional and metabolic diagnoses were the three most common complexes of diagnoses in the present study. Among the included subgroups, hypertension, cerebrovascular disease, ischemic heart disease and diabetes were found to be the physical diagnoses most frequently registered. These findings are in line with international studies of nursing home residents [40–42].

The odds of having cardiovascular diagnoses decreased in females and increased in males with increasing CDR-SOB, and the odds became significantly different for female and male residents when the severity of dementia increased (CDR-SOB > 6). Also, the odds for musculoskeletal diagnoses slightly decreased in females and increased in males with increasing CDR-SOB. Nevertheless, the odds were lower than one for both genders when CDR increased. On the contrary, the odds of having registered endocrine, nutritional and metabolic diagnoses increased with increasing CDR-SOB for both genders. We have no firm explanation for these results, but it may be related to different gender expression of dementia, physical diagnoses and pain, triggering a diagnostic review more often in males. Some cardiovascular disease presentations are commonly undiagnosed in females [64], which might partly explain the decreased odds for cardiovascular diagnoses by increasing CDR-SOB in females. Finally, it is possible that the spouses of males visit or worry more than spouses of females, producing a difference in diagnostics of disease. However, we can only speculate, and further research would be necessary.

### Strengths and limitations

The present study has significant strengths. First of all, the study is based on a large sample of individuals in nursing homes (*n* = 2893). Another strength is the use of well reputed and established scales. Demographic and health variables of potential importance for the outcome of the study were adjusted for. Additionally, GMHR was included in the post hoc analysis to evaluate if the prevalence of physical diagnoses according to the severity of dementia persisted when adjusting for physical health. However, GMHR did not influence the results significantly. Furthermore, both samples benefit from the education of nurses prior to the data collections. Finally, the study includes nursing homes from large parts of the country.

The study also had some limitations. Firstly, dementia and severity of dementia were not based on a standardized dementia investigation with neuropsychological tests. However, CDR assessment is commonly used in nursing home studies as an accepted method to identify and measure dementia [48, 65]. Secondly, a medical examination of the residents was not performed during inclusion. Diagnoses registered in the medical records were included without any further validation of their exactness [19]. In addition to this, we do not know if the diagnoses were obtained before or after admission to a nursing home. Thirdly, the data material does not distinguish between dementia subtypes, consequently, differences in comorbidity profile of individuals with vascular and neurodegenerative dementia have been left out [19, 37]. Lastly, the inclusion of nursing homes was not based on a random selection, which makes us unable to guarantee that the sample is representative for all nursing homes in Norway.

### Implications for clinical practice and future research

The present research contributes to a better understanding of the relationship between dementia and physical comorbidity, which is highly relevant due to a growing elderly population globally [7]. The importance of thoroughly and equal diagnostics among individuals with and without dementia is also emphasised. Specific guidelines for individuals with dementia and comorbid conditions are needed to reduce health care costs and improve quality of care and health outcomes. Future research should focus on physical comorbidity in nursing home residents, and explore if dementia affects the diagnostics of physical disease.

## Conclusions

In the present study, the most prevalent complexes of physical diagnoses were cardiovascular, musculoskeletal and endocrine, nutritional and metabolic diagnoses. The number of physical diagnoses registered was lower among residents with dementia than among those without. Furthermore, the odds of having cardiovascular and musculoskeletal diagnoses increased for males and decreased for females with increasing severity of dementia, in contrast to endocrine diagnoses where the odds increased for both genders. In conclusion, comorbidity and increasing severity of dementia may complicate the diagnostics of physical disease. This highlights the importance of more attention to the registration of physical diagnoses in nursing home residents with dementia.

## Abbreviations

AIC: Akaike Information Criterion
ATC: Anatomical Therapeutic Chemical
CDR: Clinical Dementia Rating
CDR-SOB: Clinical dementia rating – sum of boxes
CI: Confidence interval
COPD: Chronic obstructive pulmonary disease
CT: Computer tomography
GMHR: General medical health rating
GP: Geriatric psychiatrist
LN: Natural Logarithm
MRI: Magnetic resonance imaging
N: Number
NH: Nursing home
NP: Nurse specialized in psychiatry
NPI: Neuropsychiatric Inventory
NPS: Neuropsychiatric symptoms
OR: Odds ratio
OU: Other units
P-ADL: Personal Activities of Daily Living
PSMS: Physical Self-Maintenance Scale
Ref: Reference
REU: Rehabilitation unit
RN: Registered nurse
RU: Regular unit
SAS: Statistical analysis software
SCU: Special care unit
SD: Standard deviation
SPSS: Statistical package for the social sciences
TIA: Transient ischemic attack
